# Pteridophyte distribution of the Urals and adjacent areas: a dataset

**DOI:** 10.3897/BDJ.9.e76680

**Published:** 2021-11-25

**Authors:** Denis Melnikov, Alyona Tretyakova, Nickolay Grudanov, Olga Baranova, Stepan Senator, Albert Muldashev, Elena Podgaevskaya, Natalya Zolotareva, Nickolay Erokhin, Alex Vorobiev, Mikhail S. Knyazev, Valerii Glazunov, Olga Kapitonova, Venera Allayarova, Nikolay Naumenko, Elena Efimik, Sergey Malykh, Vera Merker, Yulia Morozyuk, Daria Burundukova, Dmitriy Shubin, Denis Shilov

**Affiliations:** 1 Komarov Botanical Institute of the Russian Academy of Sciences, St. Petersburg, Russia Komarov Botanical Institute of the Russian Academy of Sciences St. Petersburg Russia; 2 Ural Federal University named after the first President of Russia B.N.Yeltsin, Yekaterinburg, Russia Ural Federal University named after the first President of Russia B.N.Yeltsin Yekaterinburg Russia; 3 Institute Botanic Garden of the Ural Branch of the Russian Academy of Sciences, Yekaterinburg, Russia Institute Botanic Garden of the Ural Branch of the Russian Academy of Sciences Yekaterinburg Russia; 4 Tsitsin Main Botanical Garden of the Russian Academy of Sciences, Moscow, Russia Tsitsin Main Botanical Garden of the Russian Academy of Sciences Moscow Russia; 5 Ufa Institute of biology – Subdivision of the Ufa Federal Research Centre of Russian Academy of Sciences, Ufa, Russia Ufa Institute of biology – Subdivision of the Ufa Federal Research Centre of Russian Academy of Sciences Ufa Russia; 6 Institute of Plant and Animal Ecology of the Ural Branch of the Russian Academy of Sciences, Yekaterinburg, Russia Institute of Plant and Animal Ecology of the Ural Branch of the Russian Academy of Sciences Yekaterinburg Russia; 7 Institute of the problems of Northern development, Tyumen Scientific Centre Siberian Branch of the Russian Academy of Sciences, Tyumen, Russia Institute of the problems of Northern development, Tyumen Scientific Centre Siberian Branch of the Russian Academy of Sciences Tyumen Russia; 8 Tobolsk complex scientific station of the Ural Branch of the Russian Academy of Sciences, Tobolsk, Russia Tobolsk complex scientific station of the Ural Branch of the Russian Academy of Sciences Tobolsk Russia; 9 Udmurt State University, Izhevsk, Russia Udmurt State University Izhevsk Russia; 10 Perm State University, Perm, Russia Perm State University Perm Russia; 11 Botanical Garden of the Chelyabinsk State University, Chelyabinsk, Russia Botanical Garden of the Chelyabinsk State University Chelyabinsk Russia; 12 Natural Park "Reka Chusovaya", Nizhniy Tagil, Russia Natural Park "Reka Chusovaya" Nizhniy Tagil Russia; 13 Visimskiy State Reserve, Kirovgrad, Russia Visimskiy State Reserve Kirovgrad Russia

**Keywords:** dataset, data paper, occurrences, Lycopodiopsida, Polypodiopsida, Russia

## Abstract

**Background:**

Data on the species diversity and distribution of pteridophytes (lycophytes and ferns) in the Urals and adjacent areas are presented. The dataset includes 13,742 observations of two classes Lycopodiopsida and Polypodiopsida. In total, the dataset contains information on 16 families, 28 generas, 65 species, four subspecies and nine interspecies hybrids. All records are for lycophytes and ferns collected over 170 years between 1853 and 2021. The dataset presented is based on herbarium specimens, published data and field research conducted by the authors. This dataset is the first and important step towards generalising information on the current diversity and geographical distribution of pteridophytes in the Urals and adjacent areas.

**New information:**

The dataset contains 13,742 records of 65 species of pteridophytes occurrences in the Urals and adjacent territories: Udmurt Republic (42,100 km^2^); Perm Krai (160,600 km^2^); Sverdlovsk Oblast (194,800 km^2^); Chelyabinsk Oblast (87,900 km^2^); Republic of Bashkortostan (143,600 km^2^); Tyumen Oblast (160,100 km^2^); Yamalo-Nenets Autonomous Okrug (769,300 km^2^); Khanty-Mansi Autonomous Okrug (534,800km^2^) and Kurgan Oblast (71,500 km^2^). Each record includes a geographical description of the place of discovery and habitat, year of discovery, author of the finding and determination, as well as a link to a literary source (if the data were published) or the place of storage of the herbarium specimen. The presented dataset supplements the information on the occurrence of pteridophytes in the Russian Federation as a whole and clarifies their distribution in the Urals.

## Introduction

The study of the floristic diversity of territories is the basis for solving the problems of plant taxonomy, phytogeography, botanical resource science, protection of rare and endangered plant species etc. The vegetation cover is constantly changing and its study remains an important scientific direction.

The flora of the Urals and adjacent territories has been studied for a long time. Some of the first researchers of the Ural flora were J.G. Gmelin, P.S. Pallas, I.I. Lepyokhin, J.P. Falck and J.G. Georgi. The first large floristic summaries of the vegetation cover of the Urals were published in the late 19^th^ - early 20^th^ century were the works of P.N. Krylov (Krylov 1927), S.I. Korzhinsky (Korshinsky 1898), P.V. Syuzev (Syuzev 1912) and V.S. Govorukhin (Govorukhin 1937).

Currently, a huge amount of data has been accumulated on the biological diversity of the flora of the Urals. The results are summarised in the form of regional floristic summaries, such as checklists and flora keys. At the same time, the collected information is inaccessible to the majority of Russian colleagues and the international scientific community. The current global trend in biodiversity research is the use of the information technology and the principles of open science. Uploading the biodiversity data into the open international repositories would make them accessible to a wide range of specialists and help to find solutions to a wide range of problems at the regional and global level.

Our group of authors compiled a dataset on the diversity and distribution of Lycopodiophyta and Polypodiophyta of the Urals and adjacent areas and published it in the Global Biodiversity Information Facility (GBIF) as a Darwin Core Archive (Melnikov et al. 2021). The dataset was prepared in accordance with the concept of "data paper" (Penev et al. 2017) and contains information on the species composition, distribution and diversity of fern habitats in the Urals and adjacent areas. This is the first step towards the “*Flora of the Urals and adjacent areas*”, which will summarise all available information on the distribution of vascular plants in the study area and reflect modern views of taxonomists.

At present, 7526 records from the Urals and adjacent areas have been uploaded into the GBIF repository (without the published dataset): Udmurt Republic 156 records; Perm Krai 528 records; Sverdlovsk Oblast 2037 records; Chelyabinsk Oblast 743 records; Republic of Bashkortostan 779 records; Tyumen Oblast 822 records; Yamalo-Nenets Autonomous Okrug 1703 records and Khanty-Mansi Autonomous Okrug 649 records (GBIF.org 2021). Previously-published datasets for pteridophytes contain only few records and these records are very unevenly distributed over the studied region. The dataset presented by us (Melnikov et al. 2021) is the largest and includes 13,742 records and this is 63% of the records in the total published data. In addition, our dataset for the first time provides data on the distribution of lycophytes and ferns in the Kurgan Oblast. Other large datasets, which contain information on pteridophytes of the Urals are iNaturalist Research-grade Observations (4512 records, 21.9%) (Ueda 2021), Moscow University Herbarium (MW) (982 records, 4.8%) (Seregin 2021) and the Herbarium of the Institute of the Problems of Northern Development (TMN) (511 records, 2.5%) (Glazunov 2021). Our dataset contains the most complete information on the biological diversity of lycophytes and ferns in the Urals. Other datasets can be viewed as complementary. For example, thanks to the amateur researchers, *Botrychiumalaskense* W.H. Wagner & J.R. Grant and *Woodsiapulchella* Bertol were found for the first time in the studied area (Ueda 2021).

## General description

### Purpose

The main purpose of this study is the presentation of a published dataset on the distribution of lycophytes and ferns of the Urals and adjacent areas in GBIF. One of our primary goals is to make our data web-accessible for researchers.

## Project description

### Title

Flora of the Urals and adjacent areas

### Personnel

Denis Melnikov, Alyona Tretyakova, Nicolai Grudanov, Olga Baranova, Stepan Senator, Albert Muldashev, Elena Podgaevskaya, Natalya Zolotarjova, Nickolay Erokhin, Alex Vorobiev, Mikhail Knyazev, Valerii Glazunov, Olga Kapitonova, Venera Allayarova, Nikolay Naumenko, Elena Efimik, Sergey Malykh, Vera Merker, Yulia Morozyuk and Daria Burundukova.

### Study area description

The studies of the biodiversity of pteridophytes were carried out in the nine-region Ural-West Siberian sector of the Russian Federation. Some additional information for each region is given in the Geographic coverage, Description.

### Funding

This work was supported in part by the Program for Improving the Competitiveness of the Ural Federal University (the decree no. 211 of the Government of the Russian Federation, contract No. 02.A03.21.0006), the state assignments АААА-А19-119031290052-1 (Komarov Botanical Institute RAS), no. 0111-2019-0001 (Tsitsin Main Botanical Garden of the RAS), no. AAAA-A17-117072810011-1 (Institute Botanical Garden UB RAS) and no. АААА-А19-119031890084-6 (Institute of Plant and Animal Ecology UB RAS). This research was carried out within the project No. 121041600045-8 "Western Siberia in the context of Eurasian ties: man, nature, society". We thank the Ministry of Higher Education and Science of Russian Federation for the supportgiven to the Center of Collective Use “Herbarium MBG RAS” (Agreement No. 075-15-2021-678).

The work was carried out as part of the Russia 2021 project.

## Sampling methods

### Study extent

The dataset includes 13,742 observations of pteridophytes in nine administrative regions of the Russian Federation. The dataset combines three types of records: herbarium specimens, published materials and authors' field research. The earliest recorded evidence of the occurrence of Pteridophytes in the study area in historical time dates back to 1853 and the most recent to 2021. The total number of collectors for Urals flora is approximately 1600 researchers. Information about the collectors of herbarium specimens is presented in Table 1.

### Sampling description

The dataset combines three types of records: herbarium specimens, published materials and authors' field research. Authors used herbarium specimens stored in the following herbaria: Komarov Botanical Institute (LE), Tsitsin Main Botanical Garden of the Russian Academy of Sciences (MHA), the Museum of the Institute of Plant and Animal Ecology of the Ural Branch of the Russian Academy of Sciences (SVER), the Ural Federal University (UFU), the Udmurt State University (UDU), the Perm State National Research University (PERM), Chelyabinsk State University (CSUH), Tobolsk Complex Scientific Station of the UB RAS (TOB), Institute of Biology, Ufa Scientific Center of the Russian Academy of Sciences (UFA), Kurgan State University and Tyumen State University.

These data were supplemented by published materials (Kler 1914, Krylov 1927, Gorchakovsky 1950, Krasovsky and Skvortsov 1959, Igoshina 1966, Gorchakovsky 1975, Naumenko 1994, Radchenko and Fedorov 1997, Naumenko and Ivanenko 1999, Mukhin et al. 2003a, Mukhin et al. 2003b, Yudin et al. 2005, Kulikov and Kirsanova 2012).

Third data source about occurrences of lycophytes and ferns in the Urals and adjacent areas is based on field surveys performed by the authors. Every type of habitat, including natural, semi-natural and human-made in each region, was surveyed for the presence of species of pteridophytes.

### Quality control

The data were collected and processed by specialists from Komarov Botanical Institute of the Russian Academy of Sciences (RAS), Institute of Biology of the Ufa Scientific Center of the RAS, Botanical Garden of the Ural Branch of the Russian Academy of Sciences (UB RAS), Institute of Plant and Animal Ecology of the UB RAS, Ural Federal University named after the first President of Russia B. N. Yeltsin, Perm State National Research University, Udmurt State University, Tyumen Scientific Centre of the Siberian Branch of the Russian Academy of Sciences (SB RAS), Tobolsk Complex Scientific Station of the UB RAS, Botanical Garden of the Chelyabinsk State University and Tsytsin Main Moscow Botanical Garden RAS.

### Step description

The dataset preparation process included the following steps.

1. The first step of the study was to create a checklist of pteridophytes species found in the Ural and adjacent areas. The nomenclature of species was determined mainly according to Pteridophyte Phylogeny Group (Hassler 2004, PPG 2016).

2. Denis Melnikov, Alyona Tretyakova, Olga Baranova, Stepan Senator, Valerii Glazunov and Nicolai Grudanov developed a table structure that included 34 columns. Dataset fields’ names were chosen according to Darwin Core (Wieczorek et al. 2012) and include the following: "occurrenceID", "scientificName", "family", "genus", "specificEpithet", "infraspecificEpithet", "scientificNameAuthorship", "establishmentMeans","country", "countryCode", "language", "stateProvince", "county", "verbatimLocality", "decimalLatitude", "decimalLongitude", "coordinateUncertaintyInMeters", "geodeticDatum", "footprintWKT", "footprintSRS", "minimumElevationInMeters", "habitat", "eventDate", "year", "month", "day", "fieldNumber", "basisOfRecord", "recordedBy", "identifiedBy", "CollectionCode", "catalogNumber", "institutionCode", "bibliographicCitation".

3. Authors prepared tables with data on the occurrence of Pteridophytes in each of the nine Regions: Denis Melnikov and Olga Baranova – Udmurt Republic; Albert Muldashev – Republic of Bashkortostan; Mikhail Knyazev, Elena Podgaevskaya, Natalya Zolotarjova, Alyona Tretyakova and Nicolai Grudanov – Sverdlovsk Oblast; Valerii Glazunov, Olga Kapitonova and Venera Allayarova – Tyumen Oblast, Khanty-Mansi Autonomous Okrug – Yugra and Yamal-Nenets Autonomous Okrug; Nikolay Naumenko – Kurgan Oblast; Elena Efimik and Sergey Malykh – Perm Krai; Vera Merker, Yulia Morozyuk and Daria Burundukova – Chelyabinsk Oblast. Nickolay Erokhin and Alex Vorobiev provided herbarium data from the Museum of the Institute of Plant and Animal Ecology of the Ural Branch of the Russian Academy of Sciences (SVER) and Stepan Senator provided herbarium data from the Tsitsin Main Botanical Garden of the Russian Academy of Sciences (MHA) from the study regions. These datasets were combined into a “Pteridophyte of the Urals and adjacent areas” dataset.

4. Georeferencing was carried out using GPS and old samples using the Yandex-map service. All coordinates were converted into WGS84 datum. Most of the values in the fields “decimalLongitude' and 'decimalLatitute' were rounded to five decimal places. Duplicated records were deleted from the dataset.

5. Dataset “Pteridophyte of the Urals and adjacent areas” was uploaded in the GBIF repository (Melnikov et al. 2021).

## Geographic coverage

### Description

The dataset contains information on the distribution of Lycopodiophyta and Polypodiophyta in nine administrative regions of the Russian Federation including Regions of Cis-Urals (Perm Krai, the Udmurt Republic), Urals (Republic of Bashkortostan, Chelyabinsk Oblast and Sverdlovsk Oblast), and Trans-Urals and Western Siberia (Kurgan Oblast and Tyumen Oblast, Khanty-Mansi Autonomous Okrug – Yugra, Yamal-Nenets Autonomous Okrug). The largest number of occurrences (6776 or 49.3%) and species (58) were made in Sverdlovsk Oblast, while the fewest occurrences (174 or 1.3%) and species (3) were made in Kurgan Oblast (Table 2).

The Udmurt Republic and the Perm Krai are located in the Cis-Urals Region. The area of the Udmurt Republic is about 42,100 km^2^. In the north-south direction, the Region stretches across 270 km (56°00' N and 58°30' N) and in the west-east direction, 180 km (51°15'E and 54°30' E). The Perm Krai is located within 61°39′–56°06′ N and 51°47′–59°39′ E. The area of the Region is 160,600 km^2^. In the north-south direction, the Region stretches across 600 km and in the west-east direction, 400 km. The territory of Udmurtia and most of Perm Krai are situated in the north-east of the East European Plain. The relief is predominantly flat, with alternating hills and depressions. The eastern districts of the Perm Krai are situated in the foothills of the Middle and Northern Urals. The relief varies from ridgy hilly to low- and medium-hilly (Ovesnov 1997, Tuganaev 2000).

The next three Regions (Republic of Bashkortostan, Chelyabinsk Oblast and Sverdlovsk Oblast) are located within the Urals physical-geographical mountainous country. The Republic of Bashkortostan is located within 51°31′–56°34′ N and 53°10′–59°59′ E. The area of the Republic is 143,600 km^2^. In the north-south direction, the Region is 550 km long and 450 km wide in the west-east direction. The Chelyabinsk Oblast is situated between 51º57´–56º22´ N and 57º05´–63º25´ E. The area is 87,900 km^2^ and its length from the north to the south is about 490 km and from the west to the east, it is about 400 km. The Sverdlovsk Oblast area is 194,800 km^2^. In the north-south direction, the Region is 660 km long (from 56º03' N to 61º57' N) and is 560 km wide in the west-east direction (from 57º14' E to 66º11' E). Most of the territory of the Regions are located in the mountainous part of the Urals. The extreme parts of the Regions are the eastern edge of the East European Plain, which corresponds to a flat and hilly relief. The eastern part of Sverdlovsk Oblast and Chelyabinsk Oblast includes sections of the West Siberian lowlands and have a typically flat relief (Kulikov 2005, Kulikov et al. 2013).

Kurgan Oblast and Tyumen Oblast, Khanty-Mansi Autonomous Okrug – Yugra and Yamal-Nenets Autonomous Okrug are located in the Trans-Urals within the West Siberian Plain with the adjacent eastern macro-slopes of the Northern, Circumpolar and Polar Urals. The area of the Tyumen Oblast is 1,464,200 km^2^, its length from south to north is 2,100 km (55°10ʹ–77°30ʹ N) and from west to east is 1,400 km (58°50ʹ–86°00ʹ E). The Region is comprised of three independent subjects of the Russian Federation: the Tyumen Oblast (with the area of 160,100 km^2^), Khanty-Mansi Autonomous Okrug – Yugra (with the area of 534800 km^2^) and Yamal-Nenets Autonomous Okrug (with the area of 769,300 km^2^). The area of the Kurgan Oblast is 71,500 km^2^, its maximum length from the north to the south is 290 km (56°48'–54°14' N), from the west to the east is 430 km (62°06'–68°37' E). The main type of relief are plains, with elevations up to 250–300 m, located mainly along the right banks of the large rivers — Ob and Irtysh (Ogorodnov 1971, Gvozdetskiy 1973, Larin 2004, Naumenko 2008).

In general, the climate of the Urals is characterised by continentality, expressed in sharp annual fluctuations in air temperature and a moderate amount of atmospheric precipitation. As an obstacle to the movement of air masses from west to east, the Urals restrains and weakens the influence of the Atlantic Ocean on the eastern territories. Behind the Urals, there is a so-called "rain shadow": there is less precipitation here than in the Cis-Urals. The annual amount of precipitation in the plains of the Cis-Urals is 450–600 mm. In the mountains of the Northern Urals, 800–850 mm (in some places more than 1000 mm) of precipitation fall annually and the average annual amount of precipitation decreases to 450–650 mm in the Middle Urals and 300–320 mm in the South Urals. In the Trans-Urals, the annual amount of precipitation ranges from 300–350 mm in the flat southeast and north to 450–600 mm in the central part, reaching a maximum in the mountains of the Subpolar and Polar Urals is more than 700 mm (Ovesnov 1997, Tuganaev 2000, Kulikov 2005, Naumenko 2008, Kulikov et al. 2013).

Moving from the north to the south, the climate becomes warmer: the average annual temperature increases from –8°C to +2°C, the duration of snow cover decreases from 170–180 to 145–160 days, respectively. The growing season (with average daily temperatures above +5°C) increases from 60 days in the north of Tyumen Oblast and 110–120 days in the northern parts of the mountainous Urals to 160–170 days in the South Trans-Ural. The hydrothermal coefficient in the northern regions is 1.8–2.0, in the central regions, it is 1.4–1.6 and in the warmest southern regions, it varies from 0.6 to 1.1. In the Cis-Urals, the sum of positive temperatures above +10°С ranges from 1250–1300°С in the northeast to 1950–2000°С in the south-western regions. In the mountainous part and the Trans-Urals, the sum of temperatures varies from north to south from 1000–1250°C to 1400–1700°C. The highest values of the sum of temperatures are observed in the southern regions of Bashkiria and the Chelyabinsk Oblast and these are 2000–2300°C (Ogorodnov 1971, Gvozdetskiy 1973, Ovesnov 1997, Tuganaev 2000, Larin 2004, Kulikov 2005, Naumenko 2008, Kulikov et al. 2013).

The study area is located within five vegetation zones: tundra, forest-tundra, forest, forest-steppe and steppe. Forest vegetation occupies most of the studied area. In the mountainous regions, on the western slope of the Northern and Middle Urals, the most widespread dark coniferous forests are *Piceaobovata* and *Abiessibirica*, usually with a greater or lesser admixture of *Betula* sp. and sometimes *Tiliacordata* (as an undergrowth). In the northern part of the Sverdlovsk Oblast and Perm Krai, there are forests with *Pinussibirica*. In the Cis-Urals, on the western macroslope of the southern part of the Middle and northern part of the Southern Urals, there are coniferous broad-leaved forests of *Piceaobovata* and *Abiessibirica* with a more or less significant admixture of *Tiliacordata*, *Ulmusglabra* and *Acerplatanoides*. On the territory of the Republic of Bashkortostan (the southern part of the Bashkir Cis-Urals) and adjacent areas of the Chelyabinsk Oblast, broad-leaved mixed forests of *Tiliacordata*, *Ulmusglabra*, *Acerplatanoides* prevail, but areas of *Acerplatanoides* and *Quercusrobur* forests with a slight admixture of other broad-leaved species are also common. The eastern slope of the Urals and the northern part of the West Siberian Plain are territories with a continuous predominance of pine forests (*Pinussylvestris*) with an insignificant admixture of *Larixsibirica* (Ovesnov 1997, Tuganaev 2000, Kulikov 2005, Baranova and Puzyrev 2012, Kulikov et al. 2013).

In the mountains, near the upper border of the forest (in the subalpine belt), there is a strip of sparse *Piceaobovata*, *Larixsibirica* and *Pinussibirica* forests alternating with a birch krummholz formation, *Juniperussibirica* knee timbers and subalpine tall grass meadows. Above, it is a belt of mountain tundra (alpine). The mountain-tundra vegetation is dominated by shrub-lichen and grass-moss mountain tundra; other types of tundra communities (Vaccinieto-uliginosi-lichen, Salicetum, Betuletum nanae, Juncus-dominated, *Dryas* and *Arctousalpina* communities) are much less common.

Forest-steppe vegetation is represented by two areas. The first is located in the western part of the study area (the Cis-Ural forest-steppe): the Kungur forest-steppe in the Perm Krai, the Krasnoufimskaya forest-steppe in the Sverdlovsk Oblast and the Mesyagutov forest-steppe in Bashkiria and in the western part of the Chelyabinsk Oblast. The vegetation cover here is represented by a combination of birch, aspen, pine-birch and oak-birch groves on the northern slopes of hills, in ravines and depressions of the relief with motley-grass meadow steppes and steppe meadows on gentle slopes of hills and river valleys.

The second area of forest-steppe vegetation is located in the east, in the southern part of the Urals sector of the West Siberian Plain (the forest-steppe of the Trans-Urals). This territory covers the Kurgan Oblast, the east of the Chelyabinsk Oblast and Sverdlovsk Oblast and the south of the Tyumen Oblast. The vegetation consists of birch and aspen-birch ‘kolok’ (forest in the steppe), small areas of steppe pine (*Pinussylvestris*) and pine-birch (*Pinussylvestris*+*Betulaalba* s.l.) forests, alternating with areas of steppe meadows, meadows and petrophytic steppes.

The steppe zone occupies the southern part of the Trans-Urals within the Chelyabinsk Oblast and Bashkiria and is also represented in the southern part of the Bashkir Cis-Urals. The vegetation cover is represented on the watersheds by communities of true herb-feather-grass and feather-grass-fescue steppes. In hollows with more abundant moisture, there are areas of meadow steppes and steppe meadows. Petrophytic steppes are widespread along the outcrops of rocks on the tops and slopes of the ridges (Ovesnov 1997, Kulikov 2005, Naumenko 2008, Kulikov et al. 2013).

The Tyumen Oblast is one of the most water-logged regions of the world. Swamps occupy more than 50% of the area here. For the Northern, Subpolar and Polar Urals, within the boundaries of the region, a low-mountainous and, in part, a middle-mountainous relief are characteristic. At the level of 61–62°N passes the southern boundary of the distribution of permafrost. In the north of the Tyumen Oblast, there are forest-tundra and tundra vegetation, dominated by dwarf birch (*Betulanana* L.) and moss-lichen tundra (Ogorodnov 1971, Gvozdetskiy 1973, Larin 2004).

In the taiga zone, podzolic, soddy-podzolic soils and grey forest soils are the most widespread. In the steppe and forest-steppe zones, meadow-chernozem soils, leached, podzolised and typical chernozems are represented. Saline soils (salts and solonchaks) are often formed in depressions of the relief. In tundra, cold tundra-gley soils are the most widespread, in forest tundra – gley-podzol soils dominate (Ogorodnov 1971, Gvozdetskiy 1973, Ovesnov 1997, Tuganaev 2000, Larin 2004, Kulikov 2005, Naumenko 2008, Baranova and Puzyrev 2012, Kulikov et al. 2013).

### Coordinates

51.66 and 71.42 Latitude; 98.77 and 51.18 Longitude.

## Taxonomic coverage

### Description

The dataset includes 13,742 observations of two classes, Lycopodiopsida and Polypodiopsida. The dataset contains information on three families, seven genera, 12 species, two subspecies and two interspecies hybrids of Lycopodiopsida and 13 families, 21 genera, 53 species, two subspecies and seven interspecies hybrids of Polypodiopsida.

The largest number of the Pteridophyte species was recorded in Sverdlovsk oblast (58 species), in Chelyabinsk oblast (51 species) and the Republic of Bashkortostan (51 species) (Table 2).

The Class Lycopodiopsida was represented by three orders (Lycopodiales, Isoëtales and Selaginellales) and three families of (Lycopodiaceae, Isoëtaceae and Selaginellaceae), seven genera and 12 species (about 14.8% of records). The Class Polypodiopsida contained most occurrences – 85.2% (Table 3).

**Figure 1.**
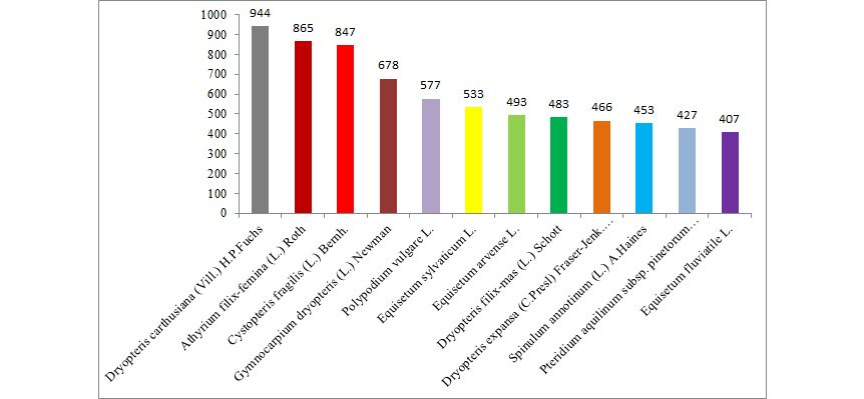
The species with the largest number of occurrences (species with more than 400 records are shown).

The obtained data provided the proper ground to identify the most common lycophytes and ferns species in the Urals. Amongst them are *Dryopteriscarthusiana* (Vill.) H.P.Fuchs, *Cystopterisfragilis* (L.) Bernh., *Athyriumfilix-femina* (L.) Roth, *Gymnocarpiumdryopteris* (L.) Newman, *Polypodiumvulgare* L. etc. (Fig. 1).

### Taxa included

**Table:** 

Rank	Scientific Name	
class	Lycopodiopsida	
order	Lycopodiales	
family	Lycopodiaceae	
order	Isoëtales	
family	Isoëtaceae	
order	Selaginellales	
family	Selaginellaceae	
class	Polypodiopsida	
subclass	Equisetidae	
order	Equisetales	
family	Equisetaceae	
subclass	Ophioglossidae	
order	Ophioglossales	
family	Ophioglossaceae	
subclass	Polypodiidae	
order	Salviniales	
family	Salviniaceae	
order	Polypodiales	
family	Pteridaceae	
family	Dennstaedtiaceae	
family	Cystopteridaceae	
family	Aspleniaceae	
family	Woodsiaceae	
family	Onocleaceae	
family	Athyriaceae	
family	Thelypteridaceae	
family	Dryopteridaceae	
family	Polypodiaceae	

## Temporal coverage

**Data range:** .

### Notes

**Figure 2.**
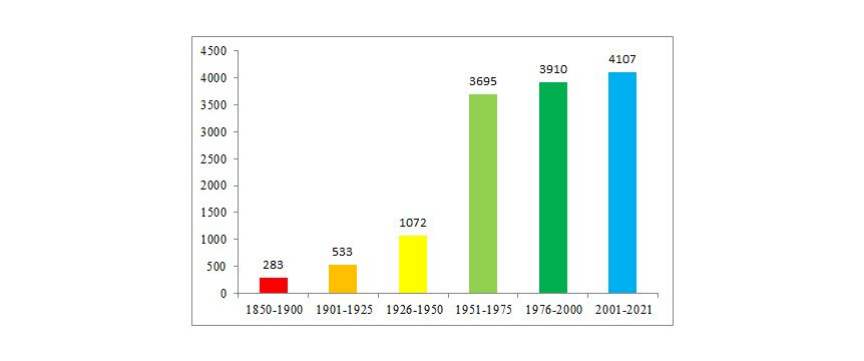
Number of occurrences in temporal scope.

The presented dataset contains information on the occurrences of lycophytes and ferns since 1853, with the most recent findings recorded in 2021 (Fig. 2). Fig. 4 shows that, in 19^th^ century and in early 20^th^ century, the number of findings of lycophytes and ferns was small. The number of occurrences increases by the second half of the 20^th^ century, with the largest number of records registered between 1951 and 2000. This result is connected with the growing interest in the study of the flora of the Urals in general and the active work conducted by the regional research institutes.

## Collection data

### Collection name

Komarov Botanical Institute (LE), Tsitsin Main Botanical Garden of the Russian Academy of Sciences (MHA), the Museum of the Institute of Plant and Animal Ecology of the Ural Branch of the Russian Academy of Sciences (SVER), the Ural Federal University (UFU), the Udmurt State University (UDU), the Perm State National Research University (PERM), Chelyabinsk State University (CSUH), Tobolsk Complex Scientific Station of the UB RAS (TOB), Institute of Biology, Ufa Scientific Center of the Russian Academy of Sciences (UFA), Kurgan State University and Tyumen State University.

### Collection identifier

LE, MHA, SVER, UFU, UDU, PERM, CSUH, TOB, UFA

### Specimen preservation method

dried

## Usage licence

### Usage licence

Creative Commons Public Domain Waiver (CC-Zero)

### IP rights notes

This work is licensed under a Creative Commons Attribution (CC-BY) 4.0 Licence.

## Data resources

### Data package title

Pteridophyte of the Urals and adjacent areas

### Resource link

https://www.gbif.org/dataset/d2875a50-0304-469d-b19e-78dc08007931

### Alternative identifiers

http://ipt.zin.ru:8080/ipt/resource?r=pteridophytes_of_the_urals

### Number of data sets

1

### Data set 1.

#### Data set name

Pteridophyte of the Urals and adjacent areas

#### Data format

Darwin Core

#### Number of columns

34

#### Character set

UTF-8

#### Download URL

https://www.gbif.org/dataset/d2875a50-0304-469d-b19e-78dc08007931

#### Data format version

7

#### Description

The presented dataset (Melnikov et al. 2021) contains 13,742 records of 65 species of pteridophytes occurrences in the Urals and adjacent territories: Udmurt Republic (1215 records, 37 species, 42,100 km^2^); Perm Krai (467 records, 22 species, 160,600 km^2^); Sverdlovsk Oblast (6776 records, 58 species, 194,800 km^2^); Chelyabinsk Oblast (1962 records, 51 species, 87,900 km^2^); Republic of Bashkortostan (1399 records, 51 species, 143,600 km^2^); Tyumen Oblast (729 records, 34 species, 160,100 km^2^); Yamalo-Nenets Autonomous Okrug (756 records, 34 species, 769,300 km^2^); Khanty-Mansi Autonomous Okrug (264 records, 36 species, 534,800km^2^) and Kurgan Oblast (174 records, 31 species, 71,500 km^2^). The dataset was compiled from herbarium specimens, published data and field research material by the authors. The presented dataset contains information on the occurrence of pteridophytes from 1853 to 2021. For each occurrence are indicated species name, locality, collection date, collector and other information.

**Data set 1.** 

Column label	Column description
occurrenceID	An identifier for the Occurrence (as opposed to a particular digital record of the occurrence). In the absence of a persistent global unique identifier, construct one from a combination of identifiers in the record that will most closely make the occurrenceID globally unique. http://rs.tdwg.org/dwc/terms/occurrenceID
scientificName	The full scientific name. http://rs.tdwg.org/dwc/terms/scientificName
family	The full scientific name of the family in which the taxon is classified. http://rs.tdwg.org/dwc/terms/family
genus	The full scientific name of the genus in which the taxon is classified. http://rs.tdwg.org/dwc/terms/genus
specificEpithet	The name of the first or species epithet of the scientific Name. http://rs.tdwg.org/dwc/terms/specificEpithet
infraspecificEpithet	The name of the lowest or terminal infraspecific epithet of the scientific Name, excluding any rank designation. http://rs.tdwg.org/dwc/terms/infraspecificEpithet
scientificNameAuthorship	The authorship information for the scientificName formatted according to the conventions of the applicable nomenclaturalCode. http://rs.tdwg.org/dwc/terms/scientificNameAuthorship
establishmentMeans	Statement about whether an organism or organisms have been introduced to a given place and time through the direct or indirect activity of modern humans. http://rs.tdwg.org/dwc/terms/establishmentMeans
country	The name of the country or major administrative unit in which the Location occurs. Included value: Russia. http://rs.tdwg.org/dwc/terms/country
countryCode	The standard code for the country in which the Location occurs. Included value: RU. http://rs.tdwg.org/dwc/terms/countryCode
language	A language of the resource. Included value: ru or en.http://purl.org/dc/terms/language
stateProvince	The name of the next smaller administrative region than country.http://rs.tdwg.org/dwc/terms/stateProvince
county	The full, unabbreviated name of the next smaller administrative region than stateProvince.http://rs.tdwg.org/dwc/terms/county
verbatimLocality	The original textual description of the place. http://rs.tdwg.org/dwc/terms/verbatimLocality
decimalLatutude	The geographic latitude (in decimal degrees, using the spatial reference system given in geodeticDatum) of the geographic centre of a Location. http://rs.tdwg.org/dwc/terms/decimalLatitude
decimalLongitude	The geographic longitude (in decimal degrees, using the spatial reference system given in geodeticDatum) of the geographic centre of a Location. http://rs.tdwg.org/dwc/terms/decimalLongitude
footprintWKT	A Well-Known Text (WKT) representation of the shape (footprint, geometry) that defines the Location. A Location may have both a point-radius representation (see decimalLatitude) and a footprint representation and they may differ from each other. http://rs.tdwg.org/dwc/terms/footprintWKT
footprintSRS	The ellipsoid, geodetic datum or spatial reference system (SRS) upon which the geometry given in footprintWKT is based. http://rs.tdwg.org/dwc/terms/footprintSRS
minimumElevationInMeters	The lower limit of the range of elevation (altitude, usually above sea level), in metres. http://rs.tdwg.org/dwc/terms/minimumElevationInMeters
habitat	A category or description of the habitat in which the Event occurred. http://rs.tdwg.org/dwc/terms/habitat
eventDate	The date-time or interval during which an Event occurred. http://rs.tdwg.org/dwc/terms/eventDate
year	The four-digit year in which the Event occurred, according to the Common Era Calendar. http://rs.tdwg.org/dwc/terms/year
month	The integer month in which the Event occurred. http://rs.tdwg.org/dwc/terms/month
day	The integer day of the month on which the Event occurred. http://rs.tdwg.org/dwc/terms/day
fieldNumber	An identifier given to the event in the field. Often serves as a link between field notes and the Event. http://rs.tdwg.org/dwc/iri/fieldNumber
basisOfRecord	The specific nature of the data record. Included value: Human Observation. http://rs.tdwg.org/dwc/terms/basisOfRecord
recordedBy	A list (concatenated and separated) of names of people, groups or organisations responsible for recording the original Occurrence. http://rs.tdwg.org/dwc/terms/recordedBy
identifiedBy	A list (concatenated and separated) of names of people, groups or organisations who assigned the Taxon to the subject. http://rs.tdwg.org/dwc/terms/identifiedBy
CollectionCode	The name, acronym, coden or initialism identifying the collection or dataset from which the record was derived. http://rs.tdwg.org/dwc/terms/collectionCode
catalogNumber	An identifier (preferably unique) for the record within the dataset or collection. http://rs.tdwg.org/dwc/terms/catalogNumber
geodeticDatum	The ellipsoid, geodetic datum, or spatial reference system (SRS) upon which the geographic coordinates given in decimalLatitude and decimalLongitude are based.http://rs.tdwg.org/dwc/iri/geodeticDatum
institutionCode	The name (or acronym) in use by the institution having custody of the object(s) or information referred to in the record.http://rs.tdwg.org/dwc/terms/institutionCode
coordinateUncertaintyInMetres	The horizontal distance (in metres) from the given decimalLatitude and decimalLongitude describing the smallest circle containing the whole of the Location. Leave the value empty if the uncertainty is unknown, cannot be estimated or is not applicable (because there are no coordinates). Zero is not a valid value for this term.http://rs.tdwg.org/dwc/terms/coordinateUncertaintyInMeters
bibliographicCitation	A bibliographic reference for the resource. http://purl.org/dc/terms/bibliographicCitation

## Additional information

Melnikov D, Tretyakova A, Grudanov N, Baranova O, Senator S, Muldashev A, Podgaevskaya E, Zolotareva N, Erokhin N, Vorobiev A, Knyazev M, Glazunov V, Kapitonova O, Allayarova V, Naumenko N, Mochalov A, Efimik E, Malykh S, Merker V, Morozyuk Y, Burundukova D, Shubin D, Shilov D (2021). Pteridophyte of the Urals and adjacent areas. Version 1.7. Komarov Botanical Institute, Russian Academy of Sciences, St. Petersburg. Occurrence dataset https://doi.org/10.15468/hatxa5 accessed via GBIF.org on 2021-10-15.

## Figures and Tables

**Table 1.** Number of records made by authors. Only authors with more than 50 records are shown.

Authors	Number of records
Storozheva M. M.	1221
Shurova E. A.	1197
Podgaevskaya E. N.	715
Zolotareva N. V.	611
Salmina N. P.	417
Muldashev A. A.	413
Knyazev M. S.	398
Kler O. Ye.	394
Baranova O. G.	376
Kulikov P. V.	328
Filroze E. M.	324
Sartakova L. I.	319
Gorchakovsky P. L.	303
Tretyakova A. S.	291
Erokhina O. V.	211
Galeeva A. Kh.	209
Pustovalova L. A.	199
Merker V. V.	193
Ivchenko T. G.	169
Morozova L. M.	169
Nikonova N. N.	166
Puzyrev A. N.	137
Khozyainova N. V.	135
Shiyatov S. G.	134
Erokhin N. G.	125
Igoshina K. N.	119
Nikitin N. A.	107
Gruner N. M.	104
Krasovsky L. I.	101
Gorbunova Zh. F.	92
Trofimova Z. I.	90
Shilov D. S.	86
Fedotova K. P.	78
Shalygin B.	78
Ektova S. N.	73
Fedorov Yu. S.	72
Kapitonova O. A.	60
Naumenko N. I	59
Helm P. G.	58
Rychkova N. N.	53
Shilova I. I.	53
Gordeev M. V.	50

**Table 2.** Number of species and occurrences in the studied regions

Regions	Number of		% of all occurrences
	species	occurrences	
Udmurt Republic	37	1215	8.8
Perm Krai	22	467	3.4
Sverdlovsk Oblast	58	6776	49.3
Chelyabinsk Oblast	51	1962	14.3
Republic of Bashkortostan	51	1399	10.2
Tyumen Oblast	34	729	5.3
Yamalo-Nenets Autonomous Okrug	34	756	5.5
Khanty-Mansi Autonomous Okrug	36	264	1.9
Kurgan Oblast	31	174	1.3

**Table 3.** Taxonomic distribution of lycophytes and ferns and species entries amongst families in the dataset.

Plant family	Number of					% of all occurrences
	genera	species	sub-species	inter species hybrid	entries	
Lycopodiopsida						
Isoëtaceae	1	2			9	0.1
Lycopodiaceae	5	9	2	2	1980	14.4
Selaginellaceae	1	1			68	0.5
Total Lycopodiopsida, 3	7	12	2	2	2057	15.0
Polypodiopsida						
Salviniaceae	1	1			25	0.2
Aspleniaceae	1	4	1		703	5.1
Athyriaceae	3	5			1361	9.9
Cystopteridaceae	2	8		1	1961	14.3
Dennstaedtiaceae	1	1	1		427	3.1
Dryopteridaceae	2	7		2	2231	16.2
Equisetaceae	1	9		3	2370	17.2
Onocleaceae	1	1			343	2.5
Ophioglossaceae	4	7			601	4.4
Polypodiaceae	1	1			577	4.2
Pteridaceae	1	2			45	0.3
Thelypteridaceae	2	2			543	4.0
Woodsiaceae	1	5		1	498	3.6
Total Polypodiopsida, 13	21	53	2	7	11685	85.0
Total Pteridophyte, 16	28	65	4	9	13742	100
